# Bone morphogenetic protein-2-mediated pain and inflammation in a rat model of posterolateral arthrodesis

**DOI:** 10.1186/s12868-016-0314-3

**Published:** 2016-12-01

**Authors:** Kendall Mitchell, Jill P. Shah, Clifton L. Dalgard, Lyubov V. Tsytsikova, Ashley C. Tipton, Anton E. Dmitriev, Aviva J. Symes

**Affiliations:** 1 Department of Pharmacology and Molecular Therapeutics, Uniformed Services University, 4301 Jones Bridge Road, Bethesda, MD 20814 USA; 2 Department of Anatomy, Physiology and Genetics, Uniformed Services University, Bethesda, MD USA; 3 Department of Surgery, Uniformed Services University, Bethesda, MD USA; 4 Department of Orthopaedic Surgery, Walter Reed National Military Medical Center, Bethesda, MD 20814 USA; 5 Division of Applied Mechanics, CDRH/OSEL, U.S. Food and Drug Administration, 10903 New Hampshire Avenue, Silver Spring, MD 20993 USA

**Keywords:** Cytokines, Hyperalgesia, Dorsal root ganglia, Inflammation, Macrophages, Bone morphogenetic protein-2

## Abstract

**Background:**

Bone morphogenetic protein-2 (BMP-2) is a pleiotropic, secreted molecule with diverse effects. The potent ability of BMP-2 to stimulate bone growth prompted its widespread clinical use for arthrodesis (spine fusion). However, elevated post-operative pain in patients treated with BMP-2 has been increasingly reported. Determining whether BMP-2 induces pain directly or whether it induces neuroinflammation, which could lower the threshold for pain, is important for developing therapeutic interventions. We therefore modeled the clinical use of BMP-2 for posterior lumbar fusion by implanting absorbable collagen sponges soaked with either recombinant human BMP-2 (rhBMP-2) or vehicle above the L4–L5 transverse processes of rat spine.

**Results:**

Using microarray analysis we found that implantation of rhBMP-2-soaked absorbable collagen sponges resulted in altered expression of numerous pro-inflammatory genes in the adjacent dorsal root ganglia (DRG) showing that implantation of rhBMP-2/absorbable collagen sponges triggers potent neuroinflammatory responses in the DRG-2. Interestingly, direct BMP-2 treatment of DRG explants resulted in changes in gene expression that were not specifically pro-inflammatory. Rats implanted with rhBMP-2 in absorbable collagen sponges also exhibited a transient change in thermal and mechanical sensitivity indicating that rhBMP-2 applied to the lumbar spine could increase pain sensitivity. Immunohistochemical analysis indicated macrophage infiltration in the DRG and spinal nerve in rats implanted with rhBMP-2/absorbable collagen sponges or absorbable collagen sponges alone, but not in rats that underwent surgery without implantation of the absorbable collagen sponges suggesting that the sponges contributed to the biological response. Indeed, analysis of DRGs taken from rats implanted with absorbable collagen sponges without rhBMP-2 showed a significant change in gene expression distinct from DRGs from rats undergoing surgery only.

**Conclusions:**

Our data indicate that implantation of rhBMP-2/absorbable collagen sponges on the lumbar spine triggers potent neuroinflammatory responses in the DRG. Importantly, however, these BMP-2 effects may be partially mediated through a response to the absorbable collagen sponges.

## Background

Bone morphogenetic proteins (BMPs) have multiple functions in growth and differentiation, including promotion of the development of cartilage and bone [1]. The osteoinductive properties of BMP-2 prompted its development into clinical use to promote bone growth and fracture healing, including arthrodesis (spine fusion). Each year, nearly 500,000 people undergo arthrodesis procedures for treating conditions of the spine. Until the development of BMP-2, arthrodesis was mainly performed using bone graft, typically harvested from the iliac crest of the same patient. FDA approval of recombinant human BMP-2 (rhBMP-2) for interbody fusion in the anterior lumbar spine was initially widely welcomed by orthopedic surgeons as it enabled more rapid spinal fusion without the morbidity associated with a second harvesting surgical site [2, 3]. Subsequently, many surgeons started performing “off-label” procedures with rhBMP-2 in all spinal regions [4]. However, increasing evidence indicates that rhBMP-2 administration is not more efficacious than bone graft [5]. Further, there are now numerous reports of side effects including ectopic bone formation, male infertility and additional neurological problems [6–9]. In 2008 the FDA issued a warning about life threatening complications associated with off-label use of rhBMP-2 in the anterior cervical spine [10]. However, rhBMP-2 is still widely used in the lumbar spine. A recent comprehensive meta-analysis of several clinical trials also revealed a significantly increased risk ratio for the development of post-operative back and leg pain in patients when rhBMP-2 is used for posterolateral arthrodesis, a surgical procedure used to fuse two or more vertebrae [5].

BMP-2 receptors, which propagate BMP-2 signaling in part by phosphorylating the BMP-2 specific transcriptional factors Smad1/5/8 [11], are expressed in nociceptive neurons of the dorsal root ganglia (DRG) and in pain processing lamina of the dorsal horn of the spinal cord [12]. While it is not known whether BMP-2 participates in nociceptive processing, the related molecules, transforming growth factor-β (TGF-β) and activin A, have prominent roles in nociception, with exogenous administration of these molecules exerting anti- or pro-nociceptive effects in a manner dependent on route of injection [12–15]. Thus, as rhBMP-2 is placed in close proximity to the DRG and spinal nerve in posterolateral arthrodesis, one possibility for the pain reported by rhBMP-2 arthrodesis patients is direct activation of nociceptors by rhBMP-2. However, it is also possible that rhBMP-2 may induce pain indirectly, for example by triggering neuroinflammation [16–21], which could lead to sensitization of nociceptors. In this paper we have modeled the off-label use of rhBMP-2, implanted within an absorbable collagen sponge (ACS) and characterized its effects on DRGs adjacent to its placement. We show that the effects of rhBMP-2 on nociception are mediated by neuroinflammatory changes, potentially enhanced by macrophage recruitment by the absorbable collagen sponge. These data suggest that some adverse effects of rhBMP-2 application are due to a combination of the cytokine and the collagen sponge.

## Methods

### Animals

All animal and care procedures were approved by the Institutional Animal Care and Use Committee of the Uniformed Services University and performed in accordance with the ARRIVE guidelines. Sasco Sprague-Dawley female rats (225–275 g), purchased from Charles River Laboratories (Frederick, MD, USA), were housed in regular cages (2 rats/cage) with access to food and water ad libitum on a 12:12 reversed light/dark cycle in USU animal facilities. Rats were allowed to acclimatize to the animal facilities for a week after arrival.

### Materials

Recombinant human bone morphogenetic protein-2 (rhBMP-2), carrier free, was purchased from R&D Systems (Minneapolis, MN, USA). Absorbable bovine type I collagen sponges (ACS; Avitene Ultrafoam) were purchased from Davol Inc (Warwick, RI, USA). rhBMP-2 was resuspended at a concentration of 100 μg/ml in vehicle (333.02 mM glycine, 25.14 mM l-glutamic acid, 1.71 mM NaCl, 0.5% (w/v) sucrose, and 0.01% (v/v) Tween 80, pH 4.5) on the day of surgery. Primers were synthesized by Integrated DNA Technologies (Coralville, IA, USA). Rat Ref-12 Expression BeadChips were purchased from Illumina (San Diego, CA, USA).

### Experimental design

Rats were randomly divided into three groups

Group 1: Surgery plus implantation with rhBMP-2 in absorbable collagen sponges (rhBMP-2/ACS)
Group 2: Surgery plus implantation with vehicle in absorbable collagen sponges (sponge or vehicle/ACS)
Group 3: Surgery alone (no sponge)

The number of animals that underwent these implantation surgeries for each of the readouts discussed in this paper are shown in Table 1. RNA was extracted from DRGs taken from all of these rats. For microarray analysis only the best quality and highest concentration four RNA samples were sent off for analysis, as the requirements for the amount of RNA and its quality were very stringent for the analysis to proceed. Behavioral analyses were conducted on a different cohort of rhBMP-2/ACS (n = 12) and vehicle/ACS (n = 11) animals. To examine changes in the DRG by immunohistochemistry, three groups of rats were sacrificed on days 7–8; rhBMP-2/ACS (n = 9), vehicle/ACS (n = 6) animals and ‘no sponge’ animals (n = 5). No animal was excluded from the study except for technical problems with the material extracted from it.

**Table 1:** Number of animals used for microarray and QPCR analyses

Post surgery days	1	3	7
Microarray			
rhBMP/2/ACS	N = 6	N = 6	N = 6
Vehicle/ACS	N = 6	N = 6	N = 6
No sponge	N = 6	N = 6	
QPCR			
rhBMP/2/ACS	N = 5	N = 7	N = 5
Vehicle/ACS	N = 5	N = 6	N = 5
No sponge	N = 5		N = 5

### Animal surgery

Ketamine/xylazine (80 and 10 mg/kg; respectively) was injected i.p. to induce general anesthesia. The backs of rats were shaved and aseptically prepared. Rats were placed in sternal recumbency on a covered heating pad and a surgical microscope was used to make a dorsal midline skin incision overlying the spinous processes from L3 to S1. The dorsal fascia and adipose tissue was dissected between L3 to S1 and the underlying muscle was cut just adjacent to the spinous process down to and near the facet joint. L4 and L5 transverse processes were exposed by retracting the overlying muscle. No decortication was performed. Two absorbable collagen sponges (ACSs) each soaked with either 400 μl of rhBMP-2 (100 μg/ml) or vehicle (333.02 mM glycine, 25.14 mM l-glutamic acid, 1.71 mM NaCl, 0.5% (w/v) sucrose, and 0.01% (v/v) Tween 80, pH 4.5) were implanted unilaterally over the L4–5 transverse processes. Thus, each animal either received 80 µg rhBMP-2 (BMP/ACS) or only vehicle (Vehicle/ACS). Additionally, a set of ‘no sponge’ animals received the identical surgery but no ACS implants. The overlying fascia and then skin of animals were closed using a 6.0 nylon suture. Animals were returned to housing facility once they recovered from anesthetic and began walking (approximately 3 h after surgery) and each animal was monitored daily.

### Behavioral testing

Behavioral testing was performed to evaluate the functional changes in the animals undergoing posterolateral arthrodesis with or without the use of rhBMP-2. Post-operative changes in nociception were evaluated using a Von Frey mechanical withdrawal test and a thermal sensitization assessment relative to baseline recordings obtained before surgery. Investigators were blinded to the group assignment of each rat, as one investigator performed the surgeries, and a second performed the behavioral measurements. All behavioral experiments were performed between the hours of 9 am and 12 pm, in a dark room.

#### Von Frey mechanical withdrawal test

Rats were placed unrestrained under plastic enclosures on an elevated wire mesh platform and allowed to acclimate for at least 30 min. Sugared cereal was given to rats during the acclimation period and occasionally during the stimulation period in order to prevent animals from falling asleep. Cereal was never given immediately before stimulation of a paw. The mechanical withdrawal threshold test was performed by applying an ascending series of calibrated nylon monofilament von Frey hairs (Ugo Basile, Italy) perpendicular to the mid-plantar surface of each animal’s hind paw at a slow, constant speed until the hair bent. A sharp withdrawal during the application of the hairs or flinching immediately upon removal of the hairs was considered a positive response. Each hair was applied to the hind paw a maximum of five times (i.e., 5 trials) unless threshold was reached. Occasionally it was observed that animals responded strongly to one hair (e.g., 80 or 100% withdrawal) and very weakly (e.g., 20%) or not at all to the next one or more successive hairs. We therefore expanded our criteria for determining mechanical threshold such that it was not based on responses to a single hair but rather when an average response of 60% was reached over three successive hairs. The logarithmic of the bend force value of the second hair in this series was established as the mechanical threshold. Series of von Frey testing was first performed on the right hind paw (ipsilateral) followed by the left hind paw. A cutoff of 60 g was used because larger filaments lift the paw, preventing accurate assessment of acute pain-induced withdrawal responses. Testing was performed for three days before surgery with the results from the final pre-surgery day used as baseline. Surgery was performed and testing was then performed on days 1, 3 and 7 post-surgery.

#### Application of thermal stimuli

Thermal testing was performed using a radiant heat stimulus from a focused incandescent light source as previously described [22]. After von Frey testing, rats were placed unrestrained under plastic enclosures on an elevated glass platform. The enclosures (23 × 13 × 13 cm) were large enough for the rats to move around. Rats were allowed to habituate for 5–10 min before testing. Care was taken to keep the glass surface dry and free of debris or excrement. Latency before paw withdrawal was taken as the readout of thermal sensitization. The intensity of the light beam was adjusted to provide a reflex withdrawal latency of approximately 6–8 s in naive animals. The mid-plantar footpad was tested twice for each hind paw. A minimum of 3 min was allowed between testing of the same paw to minimize sensitization.

### RNA isolation and quantitative PCR

Animals were sacrificed 1, 3 or 7 days post-surgery, L4 and L5 DRG and the corresponding dorsal spinal cord were removed, rinsed with PBS, quickly blotted and snap frozen on dry ice. To isolate RNA, tissue was sonicated in Trizol Reagent (Life Technologies), followed by purification with the RNeasy Mini Kit (Qiagen) according to manufacturers instructions. cDNA was synthesized from 200 ng RNA using Superscript III (Life Technologies) and QPCR performed using SYBR Green (Qiagen) in a CFX96 real time system (BioRad) with the primers listed in Table 2.

**Table 2:** Primers used for QPCR

Gene	Forward		Reverse		Amplicon	Tm (°C)
	Sequence	Position	Sequence	Position		
ATF-3	TGCCTGCAGAAGGAGTCAGAG	494–514	CTTCCGGCGTCCGCCCGTTCT	650–630	157	56
CD68	AATTCACCTGGACCTGCTCTC	837–857	ACGCAGAAGGCAATGAGCACC	1004–984	168	54
C-FOS	CTGTCAACACACAGGACTTTT	262–282	CGCTCTGGTCTGCGATGGGGC	410–390	149	50
CGRP	AACCTTAGAAAGCAGCCCAGGCATG	102–126	GTGGGCACAAAGTTGTCCTTCACCA	442–418	246	59
COL18A1	AGGTAAGGATCTGGGCTA	3064–3081	CCTCGTTGTCCGTCCCG	3237–3221	174	50
COX-2	CGAGGACTGGGCCATGGAGTG	746–766	ACCTCTCCACCGATGACCTGA	864–844	119	57
DYN	GCCCCAGCAGGAAGGGTGATA	211–231	CAGGGACGAAATCAGGGGGTT	371–359	157	57
GAPDH	ACCATCTTCCAGGAGCGAGA	292–311	GGCGGAGATGATGACCCT	438–421	147	56
ID1	GTGAGCAAGGTGGAGATACTG	304–321	GCTGGAACACATGCCGCCTCG	482–462	179	52
IL-1β	GACCCCAAAAGATTAAGGATT	136–156	AAAGAAGGTGCTTGGGTCCTC	335–315	200	50
MCP-1	CCAGAAACCAGCCAACTCTC	25–44	CCGACTCATTGGGATCATCT	216–197	192	53
S100A8	GTGCAGAATAAAAATACCGAA	159–179	GCTGTCTTTATGAGCTGCCAC	281–261	123	48
SMAD6	ACTGGATCTGTCCGATTCTAC	873–893	CCGGAGCTCCCAGTACGCCAC	1023–1003	151	50
SMAD7	AGAGTCTCCCCCTCCTCCTTA	755–775	AAGAAGTTGGGAATCTGAAAG	899–879	142	48
SUB P	AGAGGAAATCGGTGCCAAC	2128–2107	TGCCCATTAATCCAAAGAACTG	1456–1474	152	52
TNF-α	CGGTCCCAACAAGGAGGAGAA	323–343	AGGAGGGCGTTGGCACGCTGG	502–482	180	50
TRPV1	TACTACAAGGGCCAGACAGCA	672–692	GGGGCAGCTCACCAAAGTAGA	834–814	163	55

Expression of the housekeeping gene GAPDH was assessed in duplicate in parallel wells. Expression of target RNA was normalized to GAPDH, and relative changes in mRNA expression levels between control and treated samples were calculated by the delta delta threshold cycle (∆∆Ct) method as previously described [23] using BioRad CFX96 Manager software.

### Immunohistochemistry

Animals on post-operative days 7–8 were perfused with ice-cold PBS followed by 4% paraformaldehyde. Contralateral and ipsilateral DRGs (L4 and L5) were removed, post-fixed overnight in 4% paraformaldehyde and then transferred to 30% (w/v) sucrose solution for at least 48 h at 4 °C. For assessment of cell infiltration into the spinal nerve, approximately a 2 cm stretch of nerve was taken along with the corresponding L4 or L5 DRG. DRG samples were then embedded in O.C.T. and sectioned at 20 µm by cryostat. Sections were blocked with 5% normal goat serum in PBS and 0.3% Triton X-100 (TX) for 1 h. The following antisera were used: anti-GFAP, rabbit polyclonal (1:1000, DAKO Z0334); anti-ED-1, mouse monoclonal (1:500, Millipore MAB1435); and anti-IBA-1, rabbit polyclonal (1:1000, Wako, 019-19741). Fluorescent labeling with secondary antibodies was performed using Alexa Fluor 568 goat anti-mouse and Alexa Fluor 488 goat anti-rabbit (1:500, Invitrogen, A11031 and A-11034, respectively). Images were acquired using an Olympus BX61 inverted microscope attached to a Retiga EXi Aqua CCD camera using iVision software (BioVision Technologies). Cell counts of 10× images were performed using ImageJ software (National Institutes of Health). The section with the greatest number of immunopositive cells (chosen from a maximum of four per slide with inter-section intervals of 400 µm) was used to represent cell counts per animal. Cell counts were calculated as the number of immunopositive cells per unit area of tissue, as the field of view at 10× was rarely but occasionally larger than the size of the tissue.

### BeadChip genome-wide RNA expression analysis

Genome-wide RNA expression analysis was performed as previously described [24, 25]. Briefly, RNA was isolated from DRGs with the RNeasy Mini Kit (Qiagen). Total RNA integrity was determined with the RNA Nano 6000 chip on the Agilent 2100 Bioanalyzer. Total RNA with a RNA integrity number (RIN)  > 8.0 was labeled using a TotalPrep RNA Labeling Kit (Ambion) before hybridization on Rat Ref-12 Expression BeadChips (Illumina) and imaging by the Illumina iScan System at the Lerner Research Institute Genomics Core, Cleveland Clinic, Cleveland, OH. Rat Ref-12 BeadChips allow for probing of ~22,523 well-established and provisional annotated transcripts. Raw data generated from iScan imaging was outputted using the GenomeStudio software package and Gene Expression module.

### BeadArray data analyses

Raw BeadArray data was pre-processed by offset background subtraction and quantile normalization settings using the GenomeStudio data analysis software (Illumina). Post-output processing was performed by selecting transcript features if the detection *p* value <0.05 occurred in at least 75% of the samples for a experimental group (which corresponds with a 95% confidence in accurate detection over background beads). Differentially expressed BeadArray expression values between vehicle and rhBMP-2 treatment group samples were determined using the ComparativeMarker Selection module within GenePattern 3.8.0 analysis platform [26]. Differentially expressed BeadArray features were filtered for fold changes >1.66 and *p* values <0.1 between groups. Differentially expressed BeadArray features corresponding to transcripts were queried for relationships using GO, GATHER, and GSEA [27, 28]. Heatmaps of enriched transcript subsets were visualized using HeatMapViewer within GenePattern 3.8.0.

### DRG explant cultures

Excised DRG from adult naïve rats were briefly rinsed in HBSS to remove excess blood from tissue before placing in a well of 24-well plates containing DRG medium (MEM, 5% heat-inactivated horse serum, 10 mM HEPES, 1 mM Na-pyruvate, GlutaMAX (Life Technologies, NY, USA), N-2 (1×, Life Technologies) and 1× antioxidant supplement (A1345, Sigma Aldrich, MO, USA). L4 and L5 DRGs from the right side of an animal were incubated in 1 ml of DRG medium containing rhBMP-2 while the DRGs from the left side of the same animal were incubated in medium containing vehicle. After incubating for 24 h at 37 °C; 5% CO_2_, DRGs were collected and RNA isolated with Trizol. cDNA was synthesized as described and expression of different genes analyzed by QPCR. For analysis of the effect of different concentrations of BMP-2, we ran a one way ANOVA for each primer set, with Dunnetts correction for comparison to vehicle treated explants.

### Statistical analysis

For analysis of gene expression in tissue samples removed from rats that had surgery performed on one side only we performed a repeated measures two way ANOVA matching ipsilateral with contralateral samples for comparison of both treatment and side with Sidak’s correction for multiple comparisons at each time point. Counting of ED1 positive cells in DRG and nerve taken from differentially treated rats was analyzed by two way ANOVA comparing distance and treatment. For comparison of the number of ED1 positive cells in the DRG or the sciatic nerve comparing animals implanted with vehicle/ACS or surgery alone, a two tailed students *t* test was run for either the DRG or the sciatic nerve comparing the two groups. For assessment of thermal hyperalgesia or tactile hypersensitivity data were analyzed using a mixed model for repeated measures, with group (BMP2 vs. vehicle) and time (pre-op and days 1, 3 and 7) as fixed factors and a random subject effect. Separate models were estimated for each side (ipsilateral and contralateral). Pairwise comparisons between groups at each time point, and between days for each group (days 1, 3 and 7 vs. pre-op only) were examined only when the corresponding main effect or interaction term was statistically significant (*p* < 0.05). In all examinations, a *p* value of <0.05 was considered statistically significant.

## Results

**Fig. 1:**
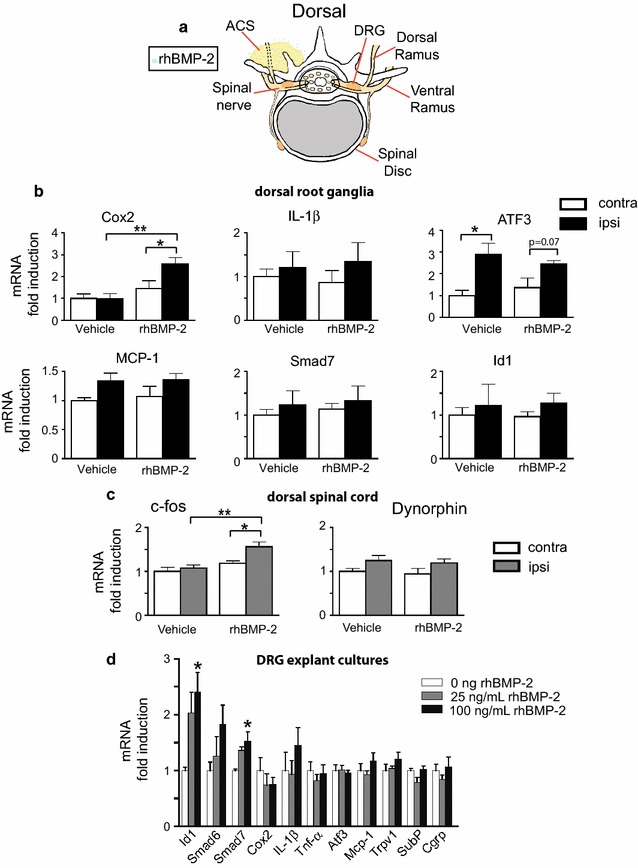
Application of rhBMP-2 in rat model of posterior lateral arthrodesis triggers changes in the PNS and CNS. **a** Illustration depicting placement of rhBMP-2/ACS next to the spine. Of the PNS structures, only the dorsal rami as well as nerves innervating the facet joint (not shown) are likely to make direct contact with rhBMP-2/ACS. **b** QPCR analysis of RNA isolated from ipsilateral and contralateral DRGs of rats 24 h after implantation of either rhBMP-2/ACS or vehicle/ACS. DRG samples were assessed using markers of inflammation (Cox-2 and IL-1β), nerve injury (ATF-3), nociceptive activity (MCP-1) and BMP-2 signaling (Smad-7 and ID-1) (mean ± SEM, n = 5, *p < 0.05, **p < 0.01). **c** QPCR analysis of RNA isolated from ipsilateral and contralateral dorsal horn spinal cord from the same animals as in 1B, 24 h after implantation of either rhBMP-2/ACS or vehicle/ACS. Increased expression of c-fos mRNA, but not dynorphin mRNA was detected in ipsilateral DSC of rhBMP-2/ACS implanted animals (mean ± SEM, n = 5*, *p* < 0.05, **p < 0.01). **d** QPCR analysis of RNA isolated from DRG explant cultures where L4 and L5 DRG from one side of a rat were combined and treated overnight in rhBMP-2 medium while L4/L5 DRG from the other side of the same animal were bathed in control medium (mean ± SEM, n = 4, *p < 0.05)

The device which the FDA approved to apply rhBMP-2 to promote lumbar spine fusion (Infuse^**®**^, Medtronic), consists of a small metallic cage containing rhBMP-2 in carrier type I bovine absorbable collagen sponge to be inserted between two vertebrae through an anterior surgical approach [2]. However, the vast majority of off-label surgical applications using rhBMP-2 for spinal fusion, either utilize a different surgical approach to the intervertebral disc (posterior approach), or directly implant the rhBMP-2 absorbed in the sponge within the posterolateral spinal space outside the intervertebral disc. Absorbable collagen sponges retain rhBMP-2, providing a half-life of rhBMP-2 in vivo of 2 days, with <5% remaining by 14 days [29]. We therefore employed a rat model for the application of rhBMP-2, by soaking absorbable collagen sponges (ACS) with either rhBMP-2 or vehicle and placing the ACS on the transverse processes across vertebrae to promote posterolateral arthrodesis. ACS soaked with either rhBMP-2 (80 µg/animal) or vehicle (control) was implanted (Fig. 1a). We picked this dose as it is a human equivalent dose (HED) of 3 mg for a 70 kg patient which is in the middle of the range of the dose of rhBMP-2 that a patient would receive [30, 31].

To determine whether implantation of rhBMP-2/ACS triggers changes in gene expression in the DRG, we measured the expression of a subset of known genes involved in inflammation, nerve injury, and BMP-2 signaling. rhBMP-2/ACS implantation resulted in a significant increase in the expression of the inflammatory marker, Cox-2, in the ipsilateral DRG compared to expression in the DRG of rats implanted with vehicle/ACS at 1 day post surgery (dps) (Fig. 1b). rhBMP-2 did not induce IL-1β, ATF-3 or MCP-1 mRNA indicating that rhBMP-2 did not globally induce inflammatory genes or those involved in nerve injury (Fig. 1b) [32–34]. However, in both groups, there was a significant induction of ATF-3 mRNA ipsilateral to surgery compared to the contralateral side. There was also no change in mRNA encoding the BMP target genes, Smad7 or ID1 mRNA.

We also examined expression of mRNA encoding the immediate early gene c-fos and the neuromodulatory peptide dynorphin in the dorsal spinal cord (DSC), as the DSC receives afferent input from the DRG. At 24 h c-fos mRNA, but not dynorphin, was significantly higher in the ipsilateral DSC of rhBMP-2 treated animals compared to expression in the ipsilateral DSC of vehicle/ACS animals (Fig. 1c). Thus, our data suggest that implantation of rhBMP-2 onto the spinal column causes changes in the DRG and these changes are transmitted to the dorsal spinal cord.

As we did not find any change in the expression of the BMP responsive genes, Smad7, Id1 or Smad6 (data not shown) in rats treated with rhBMP-2 (Fig. 1b), we wanted to determine whether cells in the DRG respond to BMP-2. We therefore treated DRG explant cultures for 24 h with different doses of BMP-2. In DRG explant cultures, rhBMP-2 induced expression of known target genes Id1 and Smad7, but did not change expression of the pro-inflammatory genes, Cox2, IL-1β or TNF-α, nor the nociceptive peptides substance P or CGRP (Fig. 1d). mRNA levels coding ATF-3, MCP-1 and the capsaicin receptor TRPV1 were also unaffected by rhBMP-2 treatment. Therefore, rhBMP-2 causes different changes in gene expression in DRG in explant culture than in innervated DRG in vivo.

**Fig. 2:**
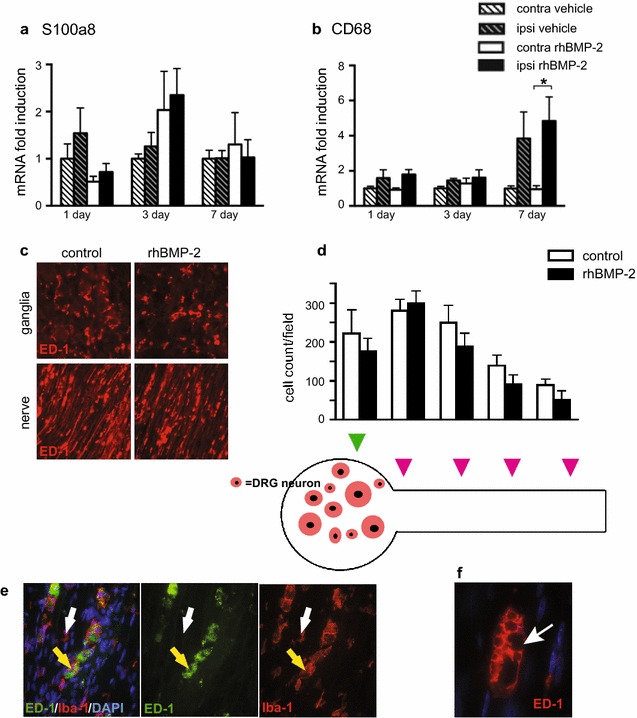
Increased numbers of macrophages in the DRG and spinal nerve of rats implanted with either rhBMP-2/ACS or vehicle/ACS. **a**, **b** QPCR analysis of RNA isolated from ipsilateral and contralateral DRGs 1, 3 or 7 days after implantation of rhBMP-2/ACS or vehicle/ACS. There was no induction of S100a8 mRNA (a neutrophil marker) at any time point, but at 7 dps, CD68 mRNA was induced ipsilateral to implantation of either rhBMP-2/ACS or vehicle/ACS (mean ± SEM, n = 5, *p < 0.05). **c** Representative immunohistochemistry image showing ED1^+^ cells 8 dps in the DRG and spinal nerve of rhBMP-2 and vehicle ACS implanted rats. **d** Number of ED1^+^ macrophages/field in rhBMP-2 or vehicle (control) rats in the DRG (*green arrowhead*), spinal nerve (the *pink arrowhead* most proximal to DRG) and ventral rami (remaining *arrowheads* depicting different locations) (mean ± SEM, n = 6 animals/group). **e**, **f** Representative double staining of spinal nerve from 7 dps rhBMP-2/ACS animals with anti-ED1 and anti-IBA1 antibodies. Images show that all ED1^+^ cells are IBA1^+^ (*yellow arrows*). There were, however, IBA1^+^ cells that were ED1^−^ (*white arrow*) and these cells were generally smaller than the ED1^+^ population. Higher magnification (× 40) reveals multiple vacuoles in ED1^+^ population of macrophages (F)

To determine whether rhBMP-2/ACS induced changes in the inflammatory profile of the DRG involved the recruitment of immune cells, we examined DRGs for the presence of macrophages and neutrophils. rhBMP-2/ACS did not induce mRNA expression of S100a8, a neutrophil marker [35], or CD68, a macrophage marker, relative to expression in vehicle/ACS implanted animals at any time point (Fig. 2a, b). We did, however, observe higher CD68 transcript levels in the ipsilateral hemisphere of both rhBMP-2 (*p* < 0.05) and vehicle groups compared to their respective contralateral controls suggesting increased presence of macrophages with surgery (Fig. 2b). Immunohistochemical analysis confirmed detection of ED1^+^ macrophages in the DRG and spinal nerve of both rhBMP-2 and vehicle groups (Fig. 2c). ED1^+^ macrophages were rarely detected near nerve fibers in naive animals (data not shown). Equal numbers of ED1^+^ macrophages were found in the spinal nerve of rhBMP-2 and vehicle treated animals on days 7–8 with a gradient of macrophages observed—higher closer to the DRG and decreasing more distal to the DRG (Fig. 2d). ED1^+^ cells throughout the nerve comprised a subpopulation of IBA1^+^ macrophages, and were generally larger than the IBA1^+^/ED1^−^ population (Fig. 2e, f). Together, these data suggest that the surgery and/or implantation of the ACS contribute to the increased macrophage presence in the spinal nerve.

**Fig. 3:**
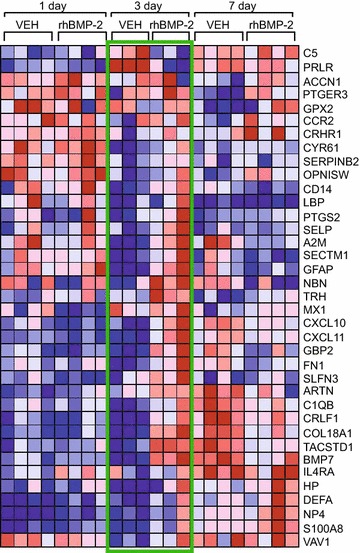
Microarray analysis indicates that rhBMP-2 induces many genes involved in neuroinflammation in the DRG. Heat map showing genes whose expression was altered at 1, 3 and 7 dps in the ipsilateral L4/L5 DRGs of rhBMP-2/ACS or vehicle/ACS implanted rats. *Blue color* intensity indicates decreasing transcript expression as compared to the mean value across all samples. *Red color* intensity indicates increasing transcript expression as compared to the mean value across all samples. The *green outlined* panel indicates high significant differences at 3 dpi. Each column represents data from RNA extracted from one rat

To assess changes in DRG that may reflect responses by resident as well as infiltrating cells following arthrodesis surgery, we performed genome-wide RNA expression profiling on samples obtained from rats implanted with either rhBMP-2/ACS or vehicle/ACS rats at post-operative day 1, 3 and 7. Analysis of expression levels between groups at each time point identified 107, 216 and 245 candidate transcripts at the 1, 3 and 7 day points respectively, with significant expression level differences between rhBMP-2 and vehicle-control treatment subjects (data not shown). A significant relationship (*p* value <0.0001 was observed between 37 of the individually annotated transcripts for functions associated with immune response (GO:0006955), response to wounding (GO:0009611) and inflammatory response (GO:0006954) using gene ontology and gene set enrichment analysis (Fig. 3). The most significant stratification between DRG samples from vehicle/ACS and rhBMP-2/ACS subjects was observed at 3 dpi (Fig. 3, center green outlined panel).

**Fig. 4:**
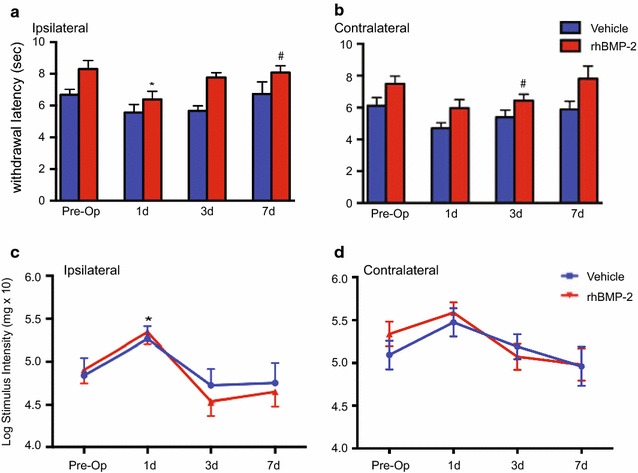
Behavioral responses of rhBMP-2/ACS or vehicle/ACS implanted rats to acute painful stimuli. Transient effects of rhBMP-2 in spinal arthrodesis model on withdrawal responses (presented as withdrawal latency) to noxious thermal stimulation (**a** ipsilateral and **b** contralateral; mean ± SEM, n = 11). Effects of rhBMP-2/ACS implantation on mechanical pain sensitivity as assessed with von Frey hair stimulation (**c** ipsilateral and **d** contralateral). *Line graph* presented as mean ± SEM of the log of the threshold von Frey hair bend force (mean ± SEM, n = 11). Statistical significance in **a**–**d** are represented as *p < 0.05, where comparisons are against pre-operative baseline for ipsilateral or contralateral or ^#^ *p* < 0.05 comparing different treatment groups at the same time point

A recent meta-analysis study reported that nearly 8 percent of patients that have undergone rhBMP-2 assisted posterolateral arthrodesis experience post-operative clinical pain [5]. To test whether the cellular and molecular changes mediated by rhBMP-2/ACS in our rodent model of posterolateral arthrodesis alters pain sensitivity, we assessed rats for development of thermal hyperalgesia and mechanical hypersensitivity. Thermal nociceptive responses of vehicle and rhBMP-2 animals were assessed by measuring the latency of ipsilateral and contralateral hind paw withdrawal from a noxious radiant heat stimulus at different time points after implantation of either rhBMP-2/ACS or vehicle/ACS (Fig. 4a, b). Baseline latency durations were acquired for each animal before surgery to provide the comparison “pre-op” value. It should be noted that baseline latency durations for the rhBMP-2/ACS animals were 1.63 (ipsilateral) and 1.38 s (contralateral) longer than vehicle/ACS animals. Statistical analysis indicated that on the ipsilateral side there were significant main effects of both group and time, but the difference between groups did not vary over time. Differences between the treatment groups were significant at 7 dps (*p* = 0.019) and approached significance at 3 dps (*p* = 0.080). Withdrawal latency changes relative to values in the same animals before the surgery (pre-op) were significant only in those implanted with rhBMP2/ACS at 1 dps (*p* = 0.018). On the contralateral side there were also significant main effects of both group and time. Differences between groups were significant at 3 dps (*p* = 0.006) and approached significance at 1 dps (*p* = 0.077). Thus, rats implanted with rhBMP-2/ACS were more sensitive to thermal stimuli at different time points after surgery, on both the ipsilateral and contralateral side.

Stimulation of hind paws with von Frey hairs was used to assess for mechanical hypersensitivity (Fig. 4c, d). Statistical analysis indicated that on the ipsilateral side there were no significant differences between BMP2 and vehicle groups at any one time point, but there was a significant main effect of time, indicating that log(force) changed over time for the BMP2 and vehicle groups combined. Indeed the rats became hyposensitive at 1 dps, followed by a hypersensitivity to this mechanical stimulation at 3 dps. Follow-up analysis indicated that the BMP-2 group showed a significant increase in log(force) on 1 dps compared to pre-op (*p* = 0.029), and a trend towards a significant decrease (hypersensitivity) at 3 dps (*p* = 0.090). On the contralateral side there was also a significant main effect of time, but none of the comparisons with pre-op reached statistical significance (1 dps vs. pre-op approached significance, *p* = 0.055), in line with a generalized hyposensitivity at 1 dps, and there were no statistical differences between treatment groups on the contralateral side. Thus, implantation with rhBMP-2/ACS led to a mild, non-significant hypersensitivity to mechanical stimuli at 3 dps.

**Fig. 5:**
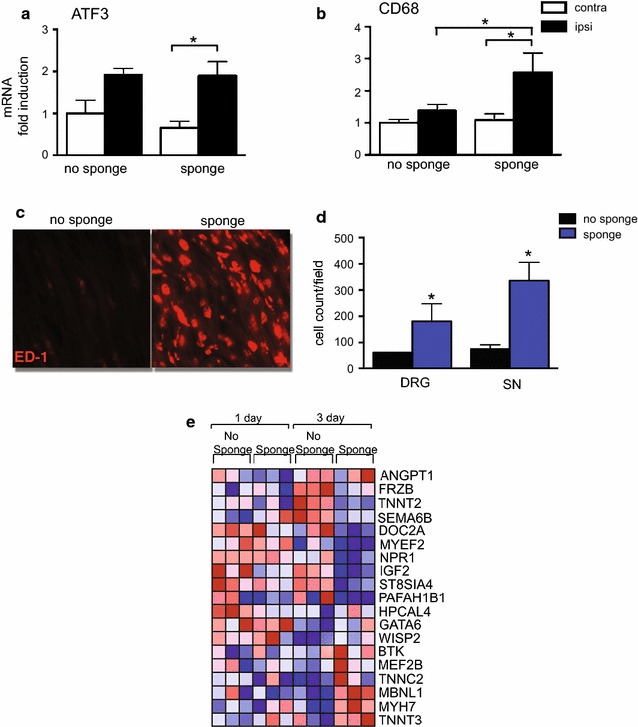
Implantation of ACS next to spine is sufficient for triggering numerous molecular and cellular changes in DRG and spinal nerve. **a**, **b** QPCR analysis of RNA isolated from ipsilateral and contralateral DRGs of rats 1 or 7 days (ATF-3 and CD68, respectively) after either implantation of vehicle/ACS (*sponge*) or the same surgical incisions but without ACS implants (*no sponge*) (mean ± SEM, n = 4 *p < 0.05). **c** Representative image of immunohistochemistry of ED1^+^ cells in the spinal nerve of rats with or without ACS implanted. **d** Cell counts of ED1^+^ macrophages in the DRG and spinal nerve of the same animals (mean ± SEM, n = 5 animals/group, *p < 0.05). **e** Heat map showing differential expression of genes in DRG at 1 and 3 dps with implantation of vehicle/ACS (*sponge*) in comparison to surgery alone (*no sponge*), as determined by bead chip analysis. *Blue color* intensity indicates decreasing transcript expression as compared to the mean value across all samples. *Red color* intensity indicates increasing transcript expression as compared to the mean value across all samples. Each column represents data from RNA extracted from one rat

The appearance of increased ED1^+^ macrophages in the DRG and spinal nerve of animals after implantation of ACS with either rhBMP-2 or vehicle suggested that the carrier ACS may have a role in recruitment of these macrophages and hence the development of inflammation. We therefore compared cellular and molecular changes in surgically incised animals after vehicle/ACS implantation with rats that underwent surgery without ACS implantation. CD68 mRNA expression trended higher in the ipsilateral DRG of animals implanted with the ACS than those with surgery alone on day 7 (Fig. 5b). Immunohistochemical analysis indicated that there were significantly greater ED-1^+^ macrophages in the ipsilateral DRG and spinal nerve of 7 days ACS implanted rats compared to the surgery only controls (Fig. 5c, d) suggesting that the presence of ACS rather than surgery alone was responsible for the increased expression of ED-1. In contrast, the nerve injury marker ATF-3 was similarly elevated in the ipsilateral DRGs of both groups compared to contralateral DRG at 24 h (Fig. 5a).

Genome-wide RNA expression analysis indicated that ACS implantation results in significant transcriptome alterations in the DRG compared to surgery alone. Clustering of genome-wide RNA expression signatures from DRG samples at 1 and 3 dps shows a complete stratification of subjects by time as well as a partial stratification of subjects by ACS presence (Fig. 5e). ACS presence was found to be associated with differential expression levels between groups for 80 candidate transcripts at 1 dps and 298 candidate transcripts at 3 dps (data not shown). The complete set of differentially expressed transcripts associated with ACS presence were observed to be significantly (*p* value 0.0002) enriched for a subset of 19 transcripts in functions in tissue morphogenesis (GO:0009653) associated with muscle development (GO: 0007517) (Fig. 5e). At 3 dps, there was strong repression of genes encoding signaling molecules such as angiopoietin1, semaphorin6B, IGF-2 and frizzled related protein and strong induction of muscle related genes such as troponin T type 3, troponin C type 2 and myosin heavy chain 7 (Fig. 5e). Interestingly, the differentially expressed transcripts associated with ACS presence was not observed to be significantly enriched for immune or inflammatory response as compared with tissue transcriptome profiling from DRGs isolated from rhBMP-2/ACS rats. These data show that implantation of the ACS alone causes significant changes in gene expression in the DRG, that are not attributable to the effects of surgery.

## Discussion

rhBMP-2 use in clinical arthrodesis is complicated with adverse side effects including pain and inflammation [5, 30]. In the present study, using a rat model of rhBMP-2 posterolateral arthrodesis, we show that rhBMP-2 induces a transient increase in thermal and mechanical sensitivity. This effect was accompanied by robust neuroinflammatory changes in and around the DRG, with some changes also transmitted to the dorsal spinal cord. We also observed that implantation of the absorbable collagen sponge alone is sufficient to cause macrophage recruitment to the DRG and spinal nerve even in the absence of rhBMP-2. Our studies suggest the inflammatory changes and altered pain sensitivity observed during rhBMP-2 arthrodesis may be mediated by a combination of effects caused by the rhBMP-2 and the ACS.

We determined that posterior lumbar fusion (PLF) surgery in rats was suitable for modeling some of the effects of rhBMP-2 on pain and inflammation. However, our procedure was different from clinical anterior or posterior lumbar *interbody* fusion surgeries (ALIF or PLIF) where the rhBMP-2/ACS is placed within a small metallic cage and then inserted into the intervertebral disc. In our model the ACS is placed directly on the transverse processes within the posterolateral spinal space outside the intervertebral disc rather than being placed within the restricted disc space, as the rodent intervertebral disc space is too small. Thus our model relates more to the off label use of rhBMP-2/ACS and does not investigate the intervertebral usage. Due to placement of the ACS the L4/L5 dorsal rami are unavoidably irritated and often cut during incision and muscle clearance. Damage to the dorsal rami may explain the ipsilateral ganglionic increase in ATF-3 with surgery, as ATF-3 induction is widely considered an indicator of nerve injury [36].

We did not detect induction of known BMP-2 responsive genes—ID1 and Smad6 in the DRGs removed from rats implanted with rhBMP-2/ACS. Instead, rhBMP-2/ACS implantation resulted in an increase in ganglionic expression of inflammatory-related genes such as Cox-2. However, direct rhBMP-2 treatment of explanted DRGs did induce ID1 and Smad7 mRNA, but not Cox-2 or other inflammatory genes. There could be several different explanations for this differential regulation of gene expression between direct rhBMP-2 treatment of DRG explant cultures, and rhBMP-2/ACS implantation-induced changes in DRG neurons in vivo. First, the effect of rhBMP-2 on DRGs in vivo could be indirect, possibly mediated by immune cells that are recruited by the presence of the implanted ACS. As the peak inflammatory response after rhBMP-2/ACS implantation at 3 dps was prior to the observed significant macrophage infiltration at 7 dps, an indirect effect through macrophages is unlikely. An indirect effect would also imply that the rhBMP-2 in the ACS was not accessible in our model to DRG neurons. Indeed, in support of a lack of direct BMP-2 signaling to DRG neurons we were unable to find any difference in pSmad1/5/8 staining between DRGs taken from rhBMP-2/ACS or vehicle/ACS rats (data not shown). Thus, the mechanism through which BMP-2 differentially alters DRG gene expression in vivo and in vitro requires further evaluation.

We detected mild transient changes in mechanical and thermal sensitivities at 1 and 3 dps (Fig. 4). This time course correlates with the increased proinflammatory gene expression at 1 and 3 dps (Fig. 3). It is widely accepted that peripheral neuroinflammation leads to sensitization of nociceptors thereby altering pain sensitivity [37]. In the DRG, inflammatory mediators including pro-inflammatory cytokines released from resident cells as wells as blood-derived immune cells can act locally to sensitize nociceptors. BMP-2 itself, although most prominently known for pro-morphogenic roles, can exert numerous pro-inflammatory effects [17, 20, 21, 38]. Administration of rhBMP-2 increases levels of circulating pro-inflammatory and osteoclastic cytokines in a rodent model of PLF [39]. Clinically, rhBMP-2 has been reported to cause life threatening cervical swelling when used for anterior cervical fusions [7] which resulted in the FDA warning against its use in this setting [10]. Also, we have previously shown that rhBMP-2 triggers potent neuroinflammatory changes after spinal cord injury [18, 40]. Therefore a generalized rapid increased inflammatory environment surrounding the DRG neurons after rhBMP-2/ACS implantation could explain the transient increase in thermal and mechanical sensitivity we found. Our data are in contrast to those of Zanella et al. [41] who find no direct effect of either rhBMP-2/ACS or ACS alone on the thermal hypersensitivity that results after direct application to the sciatic nerve in the chronic constriction injury (CCI) neuropathic pain model. However, the CCI model causes significant inflammation by itself [42], so it is not clear whether additional inflammatory stimuli would further increase the large nociceptive changes seen in this model. In our model, the increased nociception was very transient, which is largely different from the clinical scenario [30, 43]. As we showed that rhBMP-2 driven bone formation did not occur during the first 7 days after surgery, the thermal and mechanical pain we detected within the first week can not be attributed to ectopic-bone mediated nerve root compression.

The infiltration of macrophages in the DRG and spinal nerve is an indication of a robust neuroinflammatory response evoked in the present arthrodesis model. Our findings indicate that the ACS was a significant cause of infiltrating macrophages as these macrophages were detected in vehicle/ACS animals, but not in those that underwent surgery without ACS implantation (Fig. 5). Our data are in agreement with those in a recent publication that shows increased inflammatory volume following intramuscular implantation of ACS alone, that was intermediate between the edema following surgery alone, and that of rhBMP-2/ACS implantation [21]. Macrophages are well known to participate in resorption of ACS [44]. Indeed, these macrophages express ED1, a marker for phagocytizing macrophages and many contained prominent vacuoles (Fig. 5) suggestive of engulfing activities. As we did not detect large numbers of these macrophages until 7 dps, after the pain responses we monitored had returned to baseline, it is possible that these macrophages have more of a reparative role in resorption of the ACS, rather than release of pro-inflammatory cytokines that would sensitize the nociceptors. ACS implantation alone resulted in numerous other molecular changes including alterations in expression of several genes involved in tissue morphogenesis. Thus, vehicle/ACS implantation alone was not sufficient to trigger the inflammatory milieu. This pro-inflammatory environment required the rhBMP-2 and the ACS.

## Conclusions

This manuscript shows that rhBMP-2/ACS implanted onto the spine results in neuroinflammatory changes in the adjacent DRG and spinal nerve. Implantation of the ACS and exposure to rhBMP-2 resulted in distinct influences on the tissue microenvironment. An interaction of these influences resulted in neuroinflammation and an associated increase in pain sensitivity. These experiments may help explain the molecular basis for the development of radiculitis after lumbar arthrodesis and provide a rationale for the development of alternative delivery systems that could ameliorate neuroinflammatory sequelae of rhBMP-2 induced arthrodesis.

## Abbreviations

rhBMP-2: recombinant human bone morphogenetic protein -2
PLF: posterior lateral fusion
PLIF: posterior lumbar interbody fusion
ALIF: anterior lumbar interbody fusion
ACS: absorbable collagen sponge
DRG: dorsal root ganglia
Dps: days post surgery
DSC: dorsal spinal cord
CGRP: calcitonin gene related peptide
IL-1β: interleukin-1β
TGF-β: transforming growth factor-β
QPCR: quantitative PCR
